# Optimizing location of variable message signs using GPS probe vehicle data

**DOI:** 10.1371/journal.pone.0199831

**Published:** 2018-07-06

**Authors:** Lingling Fan, Liang Tang, Shaokuan Chen

**Affiliations:** 1 MOE Key Laboratory of Urban Transportation Complex System Theory and Technology, Beijing Jiaotong University, Beijing, P. R. China; 2 University of Maryland, College Park, Maryland, United States of America; Beihang University, CHINA

## Abstract

A multi-objective optimization model is proposed to allocate the location of VMSs by maximizing the average traffic guidance utility of VMSs and the number of benefited links, while minimizing information redundancy. The traffic guidance utility is defined to quantitatively measure the value of an installed VMS, which is calculated from passively collected GPS data and the physical topology of road network. The number of benefited links is to measure how many links are covered by upstream VMS to disseminate information. Information redundancy is introduced to quantify the mutual impairing between any two VMSs. A heuristic search algorithm is developed to solve the optimization model, which can calculate the saturated number of VMS for a road network and optimize the project schedule of VMS installation process based on the proposed objectives. A real-world case study is conducted in Beijing to illustrate the validity of the proposed approach, where taxis are used as probe vehicles to provide GPS data. The results show the effectiveness of the proposed multi-objective optimization model and it is promising to use the emerging GPS data to help agencies to allocate the locations of VMSs on both urban roads and highway networks, instead of relying on the subjective judgment from practitioners.

## Introduction

Advanced Intelligent Transport System (AITS) technology is an important way to balance the traffic in road network and to mitigate traffic congestion. Variable Message Sign (VMS) is one of the key components of AITS and has been applied in many cities and areas. VMS can disseminate the real-time traffic condition of downstream links to upstream drivers, which allow drivers to change their routes in advance to avoid incoming congestion or delays. How to allocate location of VMS in a road network to reach higher coverage with less devices is the key to VMS’s success.

Until now, in practice, many traffic agencies decide the VMS locations depending on subjective judgment or the experience of practitioners without quantitative analysis [1–2]. In VMS location allocation research, some published literature provides theoretical and synthetically sound work. Abbas and McCoy [3] tried to search the best location for VMS on the highway network by maximizing VMS guidance benefit using the genetic algorithm. Some studies allocate location of VMS based on simulation. Chiu et al. [4] proposed a bi-level programming model to optimally allocate location of VMS in case of an accident, in which a tabu search algorithm is assessed in the lower level by user equilibrium through simulation. Several years later, Chiu and Huynh [5] proposed another VMS location plan from the point view of cost saving under random traffic accident situations, in which dynamic traffic simulation is adopted to solve the proposed model. Zhong et al. [6] studied the effect of drivers’ compliance behavior on VMS location with simulation. Shang and Huang [7] employed cell transmission model to optimally allocate the location of VMS based on simulation. For other studies, Jeffrey M. H. [1] developed an optimization model maximizing the benefit of VMS incorporating with incident delay, driver response and dynamic traffic assignment model. Gan et al. [8] proposed a bi-level programming model to locate VMS using hypothetical data considering the interaction between VMS and traffic flow distribution in the road network. All these previous research offers valuable reference on VMS location allocation.

However, current practices in determining the VMS locations have some limitations. The VMS location allocation based on subjective judgment is not rigorous and lacks data support. Simulation-based methods may not reflect the real-world situations. Using detectors data to locate VMS is very expensive and may be hard to implement in some cities since the installation and maintenance fee of the detectors is very high to cover the whole network. In this work, a multi-objective optimization model is proposed to allocate the locations of VMSs using passively collected GPS probe data without relying on the subjective judgment from practitioners or assistance of any other additional tools.

Generally speaking, VMS should be allocated on road link where it can bring valuable information to more drivers. If the VMS facilities were too close to each other, they may not be fully utilized. Besides, it’s better to cover as many links as possible so that more people can be benefited. So a multi-objective optimization model is proposed to maximize the average traffic guidance utility and the number of benefited links while minimizing information redundancy. In the past, data limitation is one of the obstacles for the study of VMS location problem. Nowadays, with the wide spreading of GPS devices, it is much cheaper and easier to collect GPS data on the road network [9–10]. For example, in Beijing, a huge number of taxis with installed GPS devices provide tons of GPS data every day, which gives researchers the opportunity to analyze this problem using accurate, low-cost real-world data. Real-world data provide much more information of road condition in field [11]. GPS data present information for each link and continuous spatial-temporal distribution of traffic attributes in the network, while methods mentioned above do not consider all this information. To the authors’ knowledge, almost no literature uses GPS data to allocate location of VMS so far. To solve the multi-objective optimization model, a heuristic search algorithm is developed to provide a project schedule of VMS installation process and saturated number of VMS for a road network. This method can help agencies to allocate VMS location on both urban roads and highway networks, which is without relying on the assistance of any other additional tools or subjective judgment from practitioners.

The rest of the paper is organized as follows. In the next section, the concept of the guidance utility and information coverage degree are introduced, a multi-objective optimization model is proposed to allocate the locations of VMSs, then a heuristic search algorithm is developed to solve the proposed model. Section 3 conducts a real-world case study in Beijing urban road network where taxis are used as probe vehicles to provide GPS data, which illustrates the usefulness of the proposed method. Section 4 summarizes the paper and discusses some future research directions.

## Multi-objective optimization model: Methodology

In this section, a multi-objective optimization model is proposed to allocate the location of VMS by maximizing average traffic guidance utility of VMSs and number of benefited links, while minimizing information redundancy. Firstly, guidance utility is defined as the level of utilities drivers can get from the traffic information provided by VMS. Then information coverage degree is introduced to measure the information redundancy and whether a link is effected by VMS or not. A heuristic search algorithm is developed to solve the optimization model, which can calculate the saturated number of VMS for a road network and optimize the project schedule of VMS installation process based on the proposed objectives.

### Guidance utility quantification based on GPS probe data

In this subsection, guidance utility of VMS located on a link is defined as the level of utilities drivers can get from the traffic information provided by VMS, which is derived from the physical topology of the road network and GPS probe data. With the widespread use of smartphones and GPS devices, tons of GPS probe vehicle data are generated every day covering most road network, which provides a lot of valuable information for each link and continuous spatial-temporal distribution of traffic attributes for transportation planners. As GPS probe vehicle travels through a link, it will upload GPS records to the data center and provide information about the links that it passes. A GPS point record contains information like latitude, longitude, speed and timestamp. With a certain amount of probe vehicles running on the network, the traffic condition of the whole network can be monitored continuously. Through matching GPS points with the road network, traffic condition can be extracted to identify congestion.

VMS is used to disseminate its downstream information to drivers those passed the VMS spot, which allows drivers to change their route in advance to avoid incoming congestion or delays. To some extent, congestion is a good option to characterize traffic information and measured by speed information in GPS probe vehicle data. Aggregating multiple GPS data points in time interval *T* can produce a speed measure on a link. Percentile speed is used to measure speed, which can better handle the noisy speed data compared to the average speed and is widely used in scientific and field work [12]. If *n* GPS points are observed in the *m*^th^ time interval on link *i*, all the instantaneous speed data are sorted in increasing order {*v*_*k*_ (*i*,m)|*k* = 1,2,⋯⋯,*n*}. In the *m*^th^ time interval, the *p* percentile speed *v*^*p*^ (*i*,*m*) of link *i* is formulated as following [12]:

$$\left\{ \begin{array}{l} v^{p}\left(i,m\right)=v_{k}\left(i,m\right)\\ k=\left\lfloor p\times n\right\rfloor +1 \end{array}\right.$$ (1)

In above Eq (1), *p* is percentile; *i* is index number of the link; *m* means discrete time interval order; *v*^*p*^ (*i*,*m*) is the *p* percentile speed; *n* is the number of GPS data points falling on link *i* during the *m*^th^ time interval; ⌊*p*×*n*⌋ denotes the integral part of *p*×*n*. Then congestion of link *i* is identified based on speed filter. If *v*^*p*^ (*i*,*m*) is less than a speed threshold, link *i* in the *m*^th^ time interval is considered in a congestion state.

As peak hours are the toughest challenge for the road network, in this work, congestion in peak hours is chosen to evaluate traffic information for each link, in which congestion probability and duration are two important indexes to characterize congestion. After congestion identification by percentile speed, congestion probability during peak hours can be calculated. Probe vehicles GPS data during peak hours are used to measure congestion probability. For link *i*, if statistical congestion appears *d*_*i*_ times during peak hours, so the *i*th link’s congestion probability *ρ*_*i*_ can be calculated as follows [13]:

$$\rho_{i}=\frac{d_{i}}{D}\times 100\%$$ (2)

Meanwhile, based on GPS probe vehicle data, the *i*th link’s average peak hour congestion duration *t*_*i*_ can also be obtained. Both higher congestion probability and duration lead to more urgent to disseminate traffic information. So traffic information is highly correlated with congestion probability and duration. To a certain extent, traffic information *I*_*i*_ generated by link *i* is proportional to both congestion probability and duration, and suggested to be quantified as:

$$I_{i}=\rho_{i}\times t_{i}$$ (3)

In above Eq (3), *ρ*_*i*_ is the *i*th link’s congestion probability defined in Eq (2); *t*_*i*_ is the *i*th link’s average peak hour congestion duration; *I*_*i*_ is traffic information evaluated by congestion parameters on link *i* during rush hour.

As a driver passes a VMS, he or she will get information from VMS about the downstream traffic condition, which may influence the driver’s route decision. As time goes by, traffic condition may have changed when the driver arrives at congestion spot from upstream VMS location. For this reason, traffic information is prone to attenuate as time elapsed. Travel time is an ideal factor to estimate information attenuation but hard to be predicted accurately. As Wardman et al. [14] and Chatterjee et al. [15] and Si et al. [2] mentioned, instead of travel time, travel distance is thought to be better to measure information attenuation on account of its easier availability than travel time. If travel distance from VMS location to congestion spot is longer, the traffic condition is more prone to change when the driver arrives at congestion spot. In other words, the validity of information will attenuate more. Based on above analysis, travel distance is used to quantify traffic information attenuation $A_{i}^{j}$ as following [2]:

$$A_{i}^{j}=\alpha^{l_{i}^{j}},\;0<\alpha <1$$ (4)

In above Eq (4), *α* is traffic information attenuation coefficient and satisfies 0 < *α* < 1; *i* is index number of upstream link that has located a VMS; *j* is index number of the downstream link; $l_{i}^{j}$ is the shortest travel distance from link *i* to link *j*; $A_{i}^{j}$ is information attenuation as driver arrives at downstream link *j* from upstream link *i*.

**Fig 1:**
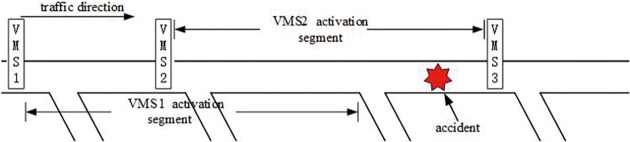
Activation segment of VMS.

Because of traffic information attenuation, each VMS has its activation segment. Usually, the activation segment is defined as VMS’s downstream segment in a certain range $l_{i}^{j}$ [5] and mathematically $A_{i}^{j}=\alpha^{l_{i}^{j}}>0$ [2], which is associated with the topology of the road network. As Fig 1 illustrated, an accident spot lies in the activation segment of VMS2, but outside the activation segment of VMS1. How many upstream VMS to be activated depends on the severity and spatiotemporal influence range of the traffic accident. The strategy for determining the spatiotemporal range of traffic information dissemination is another topic and will not be included in this work. As VMS3 is located downstream of the accident, it is useless for VMS3 to disseminate upstream traffic information. If $l_{i}^{j}$ is more than activation segment, $A_{i}^{j}$ almost approaches zero.

Traffic information generated by congestion spotted link is in need to be disseminated to upstream drivers through VMS. Accordingly, the information amount of a VMS can be quantified as cumulative traffic information of downstream links those fall in VMS’s activation segment. If VMS located on a link has more traffic information amount, this link is more valuable to locate a VMS to disseminate downstream information to drivers. Taking information attenuation into consideration, based on Eqs (3) and (4), the traffic information amount *S*_*i*_ of VMS located on link *i* can be formulated as follows:

$$S_{i}=\sum_{j\in J}\left(I_{j}\times A_{i}^{j}\right)$$ (5)

In above Eq (5), *i* is index number of a link to locate a VMS; **J** is set of downstream links that fall in the activation segment of VMS, which is closely correlated with physical topology of road network; *S*_*i*_ is traffic information amount of VMS located on link *i*; $A_{i}^{j}$ is information attenuation as driver arrives at downstream link *j* from upstream link *i* in Eq (4); *I*_*i*_ is traffic information evaluated by congestion parameters on link *i* during rush hour in Eq (3).

As traffic information is disseminated to upstream drivers, if more drivers pass a VMS to receive information, the VMS is supposed to be more effective, that is more guidance utility. If VMS is installed on link with little traffic volume or link where congestion seldom happens in its downstream segments, it cannot make many benefits. On the other hand, if more traffic information amount of VMS is generated by links in VMS’s downstream activation segment, the VMS is more effective, in other words, more guidance utility. Based on above two points, guidance utility greatly depends on traffic flow that passes VMS and information amount generated by VMS’s downstream links in activation segment. So guidance utility of VMS on a link can be defined as follows: the effect of VMS’s traffic information amount acts on drivers passed VMS spot. To a certain extent, guidance utility *G*_*i*_ is proportional to both traffic flow and the information amount of VMS located on the *i*th link, and is advised to be formulated as follows:

$$G_{i}=f_{i}\times S_{i}=f_{i}\times \sum_{j\in J}\left(I_{j}\times A_{i}^{j}\right)$$ (6)

In above Eq (6), *i* is index number of the link to locate a VMS; **J** is the set of downstream links that fall in the activation segment of VMS located on the link *i*; *f*_*i*_ is flow volume; *G*_*i*_ is guidance utility of VMS located on link *i*; *S*_*i*_ is traffic information amount of VMS located on link *i* defined in Eq (5); $A_{i}^{j}$ is information attenuation as driver arrives at downstream link *j* from upstream link *i* in Eq (4); *I*_*i*_ is traffic information evaluated by congestion parameters on link *i* during rush hour in Eq (3).

### Information coverage degree

In this subsection, information coverage degree is introduced as quantitative measurement of coverage degree of a VMS on its downstream link, which will be used to quantify information redundancy and whether a link is in benefit of upstream VMS or not. As traffic condition may have changed when drivers arrive at the downstream link from upstream VMS, information attenuation is also applicable to reckon the coverage degree of VMS on its downstream link. If a link is closer to upstream VMS, it is more effective for upstream VMS to disseminate its traffic information. Vice versa, if a link is far from upstream VMS, the effect of upstream VMS to disseminate the link’s traffic information will be lowered due to information attenuation. Based on above analysis, referring to information attenuation measured by Eq (4), information coverage degree *C*_*j*_ of link *j* can be quantified as follows:

$$C_{j}=\sum_{i\in I}A_{i}^{j}=\sum_{i\in I}\alpha^{l_{i}^{j}},\;0<\alpha <1$$ (7)

In above Eq (7), $A_{i}^{j}$ is traffic information attenuation defined in Eq (4); *α* is traffic information attenuation coefficient and satisfies 0 < *α* < 1; *i* is index number of the link located with VMS; *j* is index number of a link to measure information coverage degree; **I** is set of the *j*th link’s upstream links those have located with VMS; $l_{i}^{j}$ is the shortest travel distance from upstream link *i* to downstream link *j*; *C*_*j*_ is degree of link *j* covered by upstream VMS located on links set **I**.

### Multi-objective optimization model to allocate location of VMS

In this subsection, a multi-objective optimization model is proposed to allocate location of VMS by maximizing average traffic guidance utility of VMSs and number of benefited links, while minimizing information redundancy. For VMS’s success, VMS should be allocated on road link where it can bring valuable information to more drivers. If not considering possible information redundancy of any pair of VMS facilities, VMS should be implemented on road link with the highest guidance utility. This operation will result in maximal average guidance utility in the road network. For a VMS installed road network, let *b*_*i*_ representing whether a VMS is installed on link *i* or not, if the *i*^th^ link locates a VMS *b*_*i*_ = 1; otherwise *b*_*i*_ = 0. Average guidance utility of VMSs in the road network is defined as follows:

$$\sum_{i}G_{i}b_{i}/\sum_{i}b_{i},\;i\in N$$ (8)

In above Eq (8), *i* is index number of the link; *G*_*i*_ is guidance utility of VMS located on link *i*, which is defined in Eq (6); **N** is set of links in the road network. Therefore, the first objective is to maximize average guidance utility in the road network: $\max\;\sum_{i}G_{i}b_{i}/\sum_{i}b_{i},\;i\in N$.

However, any pair of VMS facilities with largest guidance utility may be close to each other. In this condition, if drivers have already gotten information from upstream VMS, the information provided by the current link may not as useful as the upstream one. We may waste money installing two close VMSs to provide people with redundant information. Besides, it is difficult to guarantee consistency of traffic information disseminated by two close VMSs in the road network. When drivers consecutively pass two close VMSs, if these two neighboring VMSs are close to each other and don’t provide consistent information, drivers will be confused, and it will impair the credibility of VMS. From above analysis, it can be concluded that information redundancy is caused by interaction between any two VMSs those are close to each other, which impairs the best use of VMS and is seen as resource waste. If $l_{i}^{j}=0$, the *C*_*j*_ = 1, which means information coverage is 1 for a link with VMS location on itself. Thus, if a link located with VMS has *C*_*j*_ > 1, the part *C*_*j*_ −1 comes from other VMS, which means redundancy. Information redundancy in a road network is quantified as:

$$\sum_{i}(C_{i}-1)b_{i},\;i\in N$$ (9)

In above Eq (9), *i* is index number of the link; *C*_*i*_ is information degree of link *i* defined in Eq (7); *b*_*i*_ representing whether a VMS is installed on link *i* or not, if the *i*^th^ link locates a VMS *b*_*i*_ = 1, otherwise *b*_*i*_ = 0; **N** is set of links in the road network. Physically, minimize information redundancy means maximizing the use of each VMS and minimizing the saturated number of VMS facilities in a road network. In other words, minimizing implementation cost, while providing as much information as possible to the public. Then, if road network has reached saturation with VMSs, any new added VMS will cause information redundancy. So, minimizing implementation cost can be achieved by minimizing information redundancy without artificially set budgetary constraints. Consequently, the second optimization objective is to minimize information redundancy: $\min\;\sum_{i}(C_{i}-1)b_{i},\;i\in N$.

With the increase of travel distance $l_{i}^{j}$ from VMS location on link *i* to downstream link *j*, coverage degree *C*_*j*_ decreases. When *C*_*j*_ > 0, link *j* is under benefit of upstream VMS; When *C*_*j*_ ≈ 0, link *j* is not under benefit of upstream VMS. Note *u*_*i*_ as a bivariate variable to represent whether the *i*^th^ link is under benefit of upstream VMS or not, then

$$u_{j}=\left\{ \begin{array}{ll} 1, & \mathrm{if}\;C_{j}>0\\ 0, & \mathrm{if}\;C_{j}=0 \end{array}\right.$$ (10)

In above Eq (10), *j* is index number of the link; *C*_*j*_ is the degree of link *j* covered by upstream VMS, which is defined in Eq (7). As congestion can happen on any link, ideally, information of any link should be effectively disseminated to upstream drivers through VMS. In other words, as many links as possible are expected to be covered by upstream VMS. So, the last optimization objective maximizes the number of benefited links of VMS: $\max\;\sum_{j}u_{j},\;j\in N$.

According to above analysis, the multi-objective optimization model can be formulated as follows:

$$\left\{ \begin{array}{l} \max\;\sum_{i}G_{i}b_{i}/\sum_{i}b_{i},\;i\in N\\ \min\;\sum_{i}(C_{i}-1)b_{i},\;i\in N\\ \max\;\sum_{j}u_{j},\;j\in N \end{array}\right.$$ (11)

In above Eq (11), $\max\;\sum_{i}G_{i}b_{i}/\sum_{i}b_{i}$ represents the first optimization objective to maximize average guidance utility of VMSs in the road network, in which *G*_*i*_ is the guidance utility of VMS located on link *i*, referring to Eq (6); *b*_*i*_ means if the *i*^th^ link locates with a VMS *b*_*i*_ = 1, otherwise *b*_*i*_ = 0. $\min\;\sum_{i}(C_{i}-1)b_{i}$ stands for the second optimization objective minimizing information redundancy, which can also achieve minimizing implementation cost; *C*_*i*_ is the information coverage degree of link *i* benefited from upstream VMS, referring to Eq (7). $\max\;\sum_{j}u_{j}$ demonstrates the third optimization objective to maximize number of benefited links which are under benefit of upstream VMS, in which *u*_*j*_ is defined in Eq (10).

### Heuristic search algorithm to solve multi-objective optimization model

In the proposed multi-objective optimization model, there are several nonlinear terms. The first optimization objective $\max\;\sum_{i}G_{i}b_{i}/\sum_{i}b_{i}$ is non-linear. Besides, if substituting Eq (7) of information coverage degree $C_{j}=\sum_{i\in I}A_{i}^{j}=\sum_{i\in I}\partial^{l_{i}^{j}}=\sum_{i\in N}\left(\partial^{l_{i}^{j}}\times b_{i}\right)$ into the second optimization objective $\min\;\sum_{i}(C_{i}-1)b_{i}$, it will be a non-linear expression $\sum_{i}\left[\sum_{j\in N}\left(\partial^{l_{j}^{i}}\times b_{j}\right)-1\right]b_{i}$. Heuristic algorithm has been widely used to solve non-linear optimization problems [16]. In this section, an heuristic search algorithm is developed to solve this Multi-objective Optimization Model.

Ideally, an algorithm that can give installation precedence order is a consideration if financial support is not sufficient to cover all the VMS. Another appeal is to develop an algorithm that can give the saturated number of VMS in a road network to avoid traffic information redundancy and cost waste. In this condition, a heuristic algorithm is developed to solve the proposed model and reasonably allocate VMS location, which also provides a project schedule of VMS installation process and saturated number of VMS for a road network.

**Fig 2:**
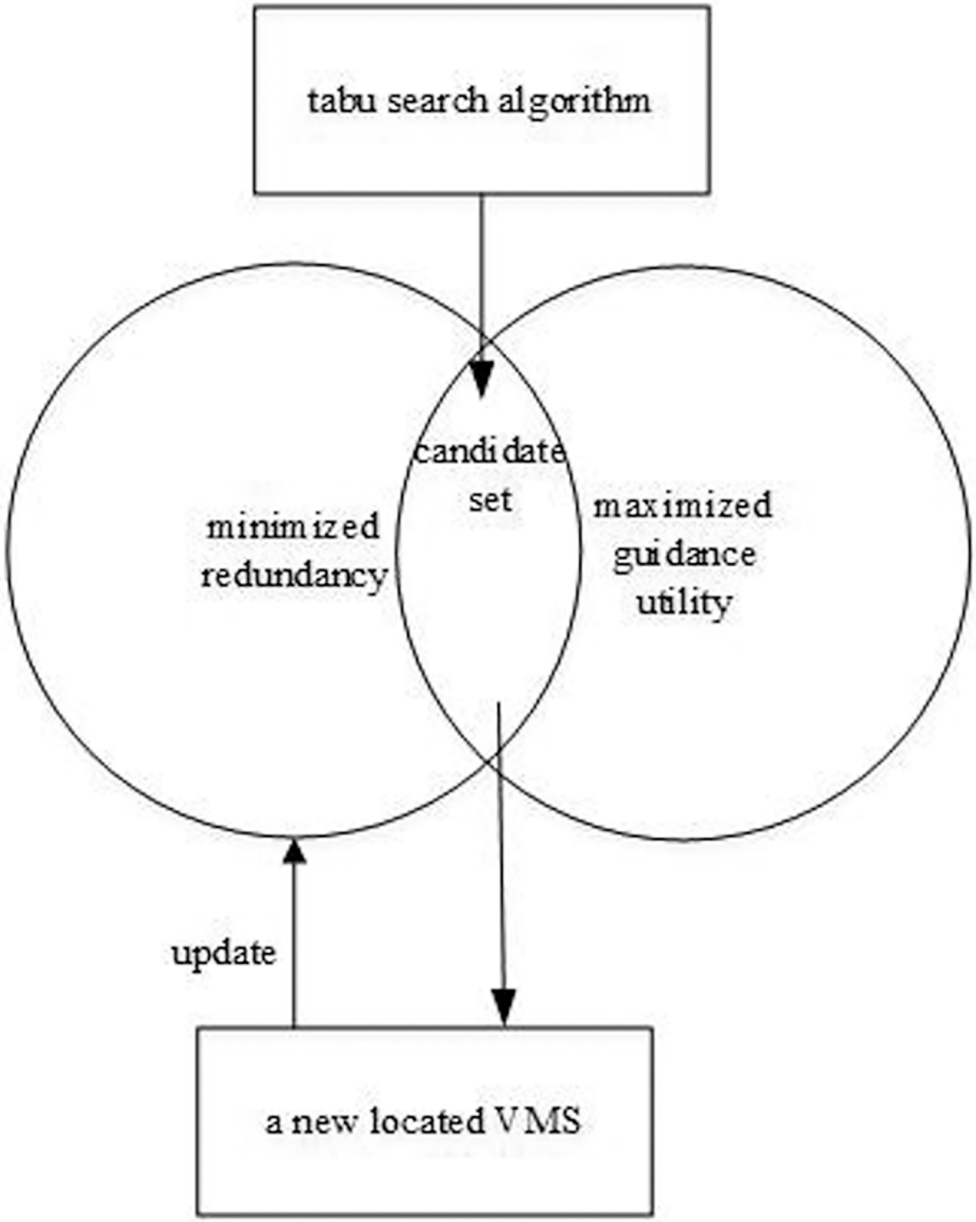
The general framework of the algorithm.

For the first optimization objective maximizing average guidance utility of VMS in the road network, VMS are intuitively located on links with the largest guidance utility. Naturally, links with largest guidance utility will be chosen to locate VMS. However, considering the second objective of minimizing traffic information redundancy, links with minimal information coverage degree but maximal guidance utility will be candidate set for locating VMS. As the third objective is to maximize the number of benefited links under coverage of upstream VMS, the candidate link is not necessarily the best option. Then tabu search algorithm is used to look through if there is a better choice in the neighborhood of the candidate link to allocate next VMS in the road network. To more clearly illustrate the algorithm, Fig 2 is used to show the general framework of the algorithm.

**Fig 3:**
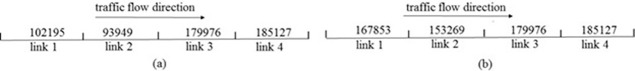
Move direction for the tabu search algorithm (the assumed value above links indicates guidance utility if VMS located on that link): (a) two consecutive links have similar large guidance utility; (b) several consecutive links have similar large guidance utility.

Tabu search algorithm is developed by Fred Glover [17–19] and a heuristic search method widely used in mathematical optimization. The algorithm starts with an initial feasible solution and chooses a series of specific search direction as the temptation. In this work, the candidate link in above paragraph is seen as an initial solution. The move direction for the search of the neighboring candidate links is illustrated in Fig 3, in which link 4 is candidate link. In Fig 3(A), link 3 and link 4 are classified into one class; link 1 and link 2 are classified into another class. If locating VMS on link 3, link 4 is under benefit of VMS through Eq (10). However, if VMS is located on link 4, link 3 is not under benefit of VMS, i.e. link 3 is not a benefited link. If link 3 and link 4 are allocated VMS respectively, these two VMSs are close to each other to cause information redundancy, which is contrary to the second optimization objective. If VMS located on link 3, the guidance utility contribution of link 3 is similar to link 4 for the road network to satisfy the first optimization objective. What’s more, both link 3 and link 4 are under benefit of VMS to fulfill the third optimization objective. Based on above analysis, in Fig 3(A), link 3 is more reasonable to install a VMS. In Fig 3(B), guidance utility of all the four links are classified into one class, which means any one of them with VMS location has similar guidance utility contribution for the road network. To avoid information redundancy and maximize the best use of each VMS, the most intuitive and feasible method is to keep distance between any pair of VMSs. If the total distance from link 1 to link 4 exceeds a certain threshold that is enough to avoid information redundancy, link 4 is chosen to install a VMS firstly. Then as algorithm continues, some upstream link of link 4 will be chosen to locate another VMS. If the distance from link 1 to link 4 is less than the threshold, according to the analysis in Fig 3(A), only link 1 is chosen to install a VMS, and all the links are under benefit of VMS located on link 1. As tabu search algorithm ends, the location of next VMS is determined.

As long as a new VMS is located in the road network, traffic information coverage *C*_*i*_ of each link will be updated according to Eq (7). Using K-NN clustering algorithm, the information coverage set **C** is classified into *b* classes. If the minimum mean value of the class in **C** is less than a preset threshold, the search is continued to locate another VMS. Otherwise, the heuristic search algorithm stops, which means the road network has reached saturation with VMSs and any new added VMS will cause information redundancy. This is to minimize implementation cost, in other words, the saturated amount of VMS in the last objective.

**Fig 4:**
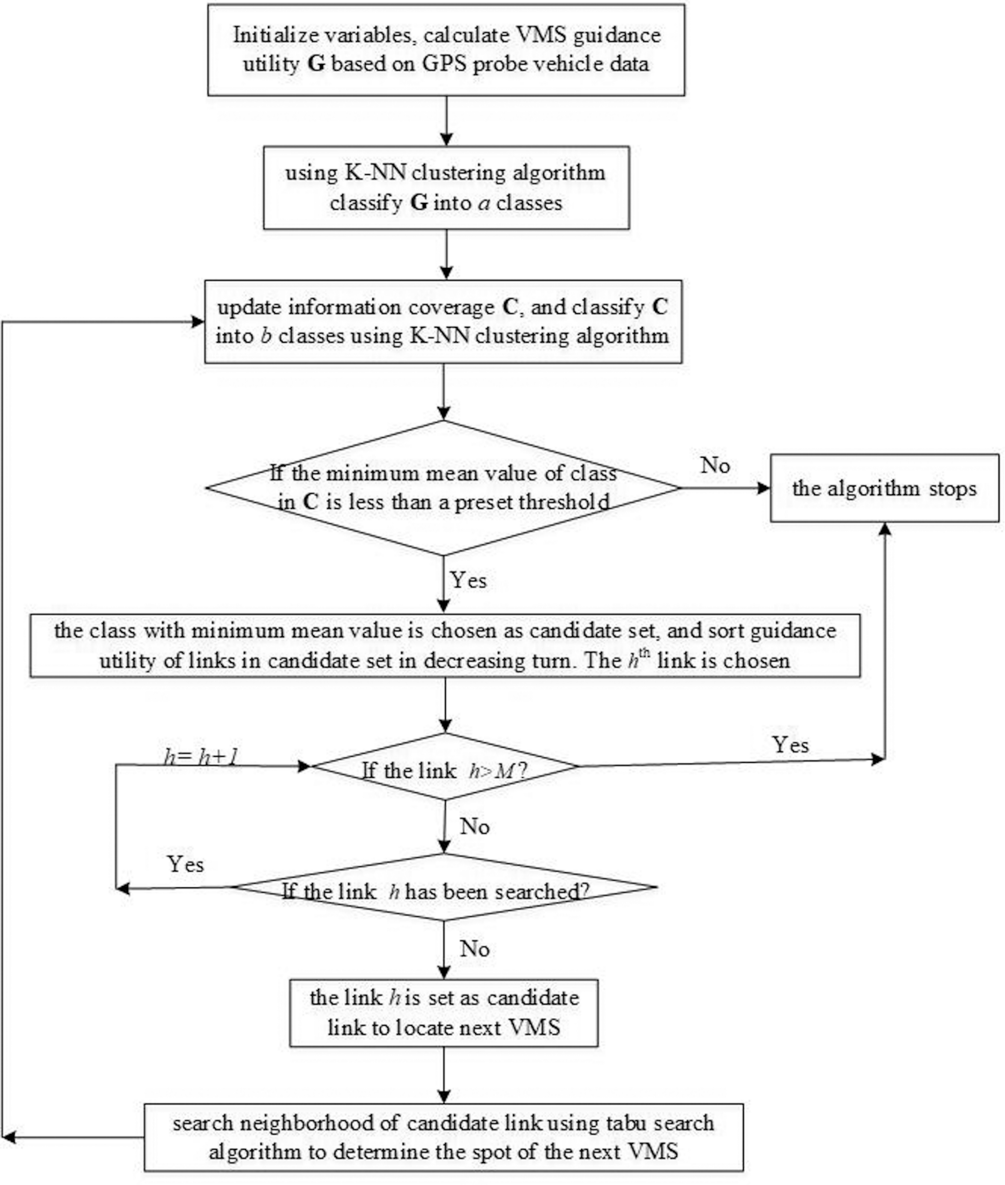
Flowchart of the heuristic search algorithm.

Fig 4 shows the flowchart of the heuristic search algorithm, which contains the following steps:

**Step 1:** Initialize variables. Links set with VMS location in the road network is **A** = *ϕ*; links set that has been searched is **S** = *ϕ*.
**Step 2:** Set traversal iteration *k* = 1 and conduct the following two steps:
        **Step 2–1:** Calculate VMS guidance utility *G*_*i*_ for each link based on GPS probe data and the topology properties of road network according to Eq (6);
        **Step 2–2:** Classify guidance utility **G** into *a* classes using K-NN clustering algorithm.
**Step 3:** Update information coverage *C*_*i*_ of each link according to Eq (7), and classify information coverage set **C** into *b* classes using K-NN clustering algorithm. The mean value of each class is calculated and the minimum mean value is represented as *mmv*. Conduct the following two steps:
        **Step 3–1:** If the minimum mean value *mmv* is less than a preset threshold, choose the information coverage class with minimum mean value as candidate set, and the number of elements in the candidate set is *M*. In this candidate set, sort links’ guidance utility in descending order. Then set the traversal iteration *h* = 1 and go to Step 4;
        **Step 3–2:** If the minimum mean value *mmv* is greater than a preset threshold, the algorithm stops.
**Step 4:** For the value *h*, conduct the following two steps:
        **Step 4–1:** If *h* ≤ *M*, choose the *h*^th^ link as exploration link and go to Step 5;
        **Step 4–2:** If *h* > *M*, the algorithm stops.
**Step 5:** For the chosen *h*^th^ exploration link, conduct the following two steps:
        **Step 5–1:** If the *h*^th^ link is not in set **S**, choose the *h*^th^ link as candidate link and go to Step 6;
        **Step 5–2:** If the *h*^th^ link is in set **S**, set *h* = *h*+1 and return to Step 4.
**Step 6:** Tabu search algorithm is conducted to search the neighborhood of the candidate link according to move direction illustrated in Fig 3. Conduct the following two steps:
        **Step 6–1:** If there is no better option in the neighborhood of candidate link, the candidate link is chosen as the final link to install the *k*^*th*^ VMS;
        **Step 6–2:** Otherwise, a better link in the neighborhood is selected as the final link to install the *k*^*th*^ VMS.

Add the final link and the searched neighborhood links to set **S**, and add the final link to set **A**. Then set *k* = *k*+1 and return to Step 3.

As heuristic search algorithm stops, a project schedule of the VMS installation process and the saturated number of VMS for a road network will be automatically output. This method can use the proposed multi-objective optimization model and the emerging GPS data to help agencies to allocate location of VMS on both urban roads and highway networks, without relying on the assistance of any other additional tools or subjective judgment from practitioners.

## A case of Beijing urban roads to locate VMS

In this section, the proposed multi-objective optimization model is tested in Beijing urban road network. Firstly, Beijing is one of the most congested cities in the world [20]. A report from AutoNavi Software Co. (a mobile web-mapping service provider) shows that on average, a driving commuter in Beijing spends 32 minutes in traffic congestion per hour during peak hours, which ranks the highest in China [21]. Besides, Variable Message Sign (VMS) has been applied in Beijing, which provides a chance to compare the VMS locations calculated by the proposed method with the ones deployed in real-world. Lastly, most of the taxis in Beijing have GPS devices installed, which provide tons of passively collected GPS data everyday.

### Data description and matching on test area map

**Fig 5:**
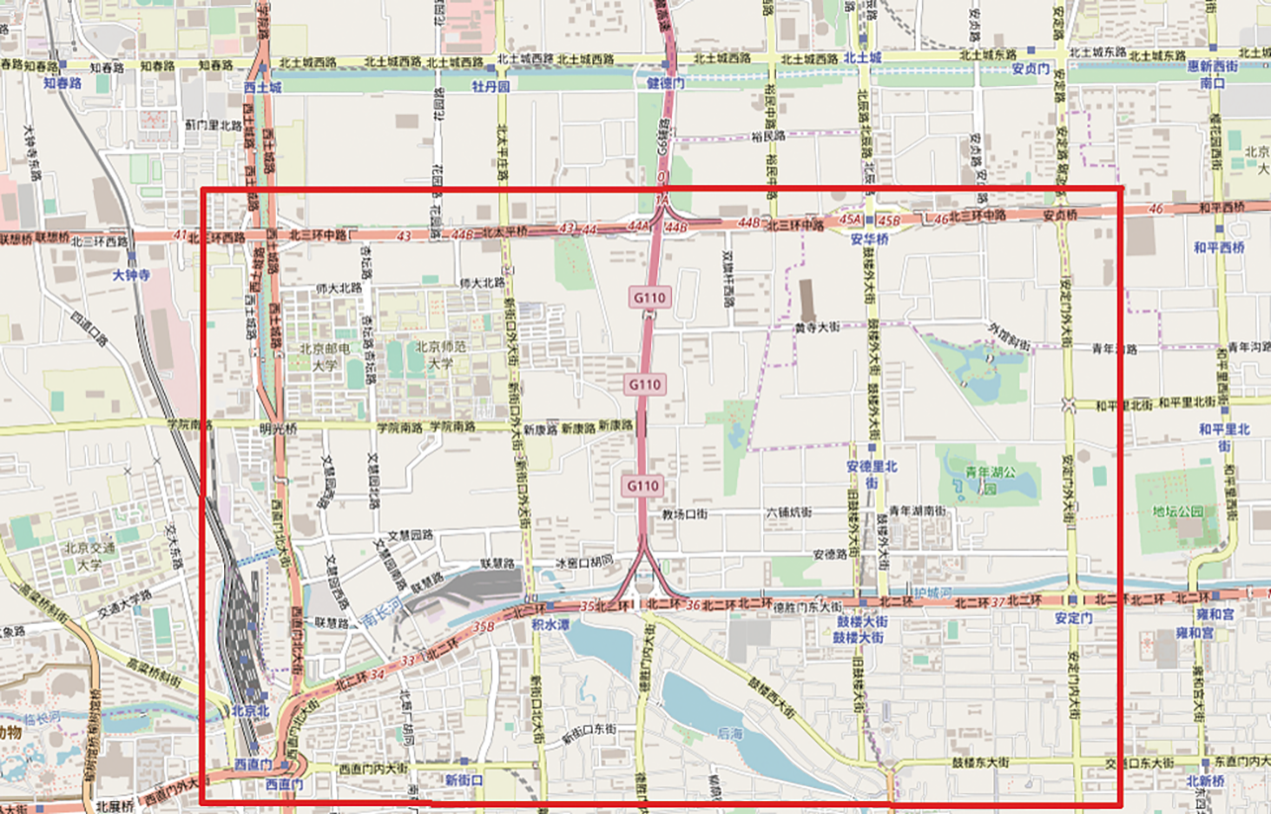
Test area in Beijing urban road OpenStreetMap contributors (openstreetmap.org).

Beijing is a giant city suffers from congestion every day and the population has reached about 21.5 million by 2015 [20]. Taxis with GPS devices are served as probe vehicles and capable of collecting and uploading GPS data to data center per 30s to 60s. There are about 46,765 taxis in Beijing [20], which provide about 56 million rows data per day in 2015. Since most roads are not blocked by buildings in Beijing, the accuracy of the GPS data are high for most of the road network. The GPS data include timestamp, position, speed, moving direction and carrying passengers or not. Only the GPS data from taxis that were carrying passengers are taken into account in this study because these taxis travel as regular vehicles in road network while uploading GPS information to data center in short time interval. The urban area of Beijing is enclosed by Ring 2–5 expressways and linked by trunks. The study area is chosen between north ring 2 and north ring 3 roads as shown in Fig 5. The north ring 2 and 3 roads are linked by some trunks.

**Fig 6:**
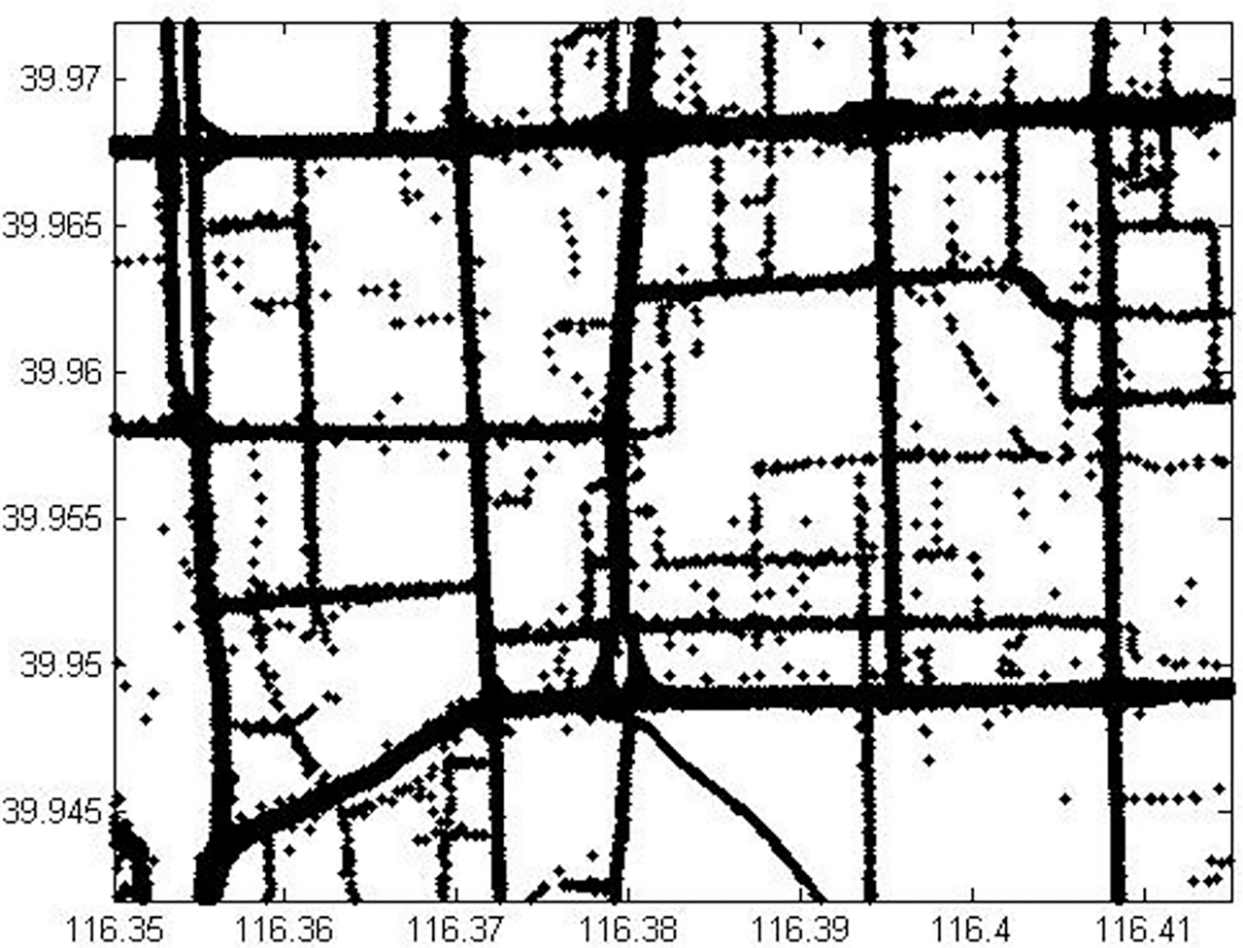
GPS data instantaneous positions during 8:00am-9:00am on Jan. 12^th^, 2015 in study area.

**Fig 7:**
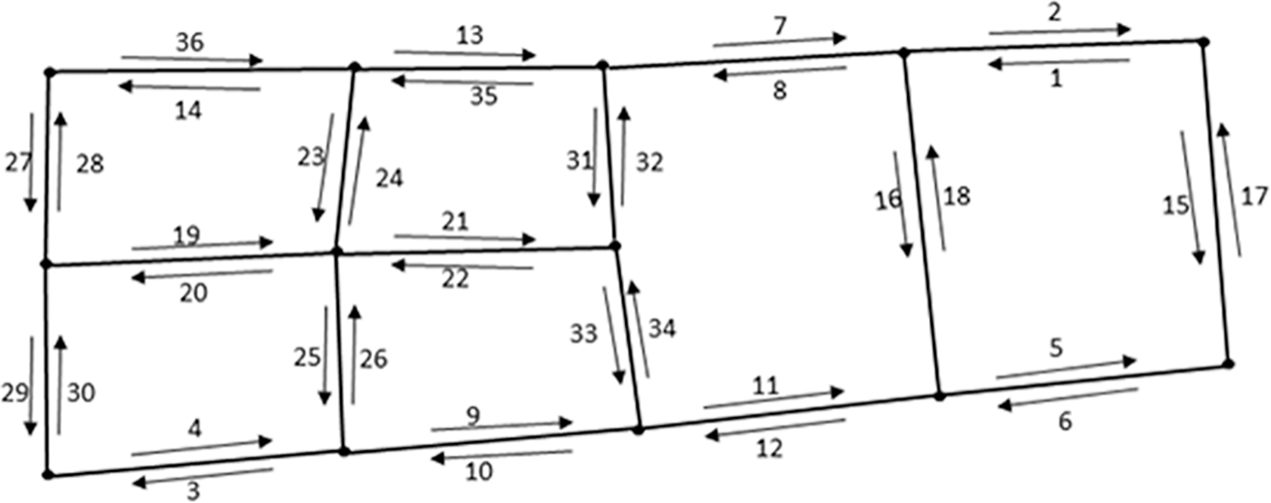
Directional links extracted from the study area (number under or above each link is index).

In this study, software ArcGIS 10.0 is used to match GPS points with study area map. During matching, data cleaning is necessary to filter out noisy GPS data. The data that falls within a certain radius of the road and has same moving direction with road are chosen for our case study. In consideration of GPS data accuracy, it is suggested to adopt appropriate radius to distinguish data for a road. Referring to reference [13], the data fell in range of a specified 30 meters radius of a link are kept, in which GPS points not on the road are filtered out. Because a road contains two links with traffic flow in two opposite directions, the two opposite direction links in a road need to be distinguished from each other in advance. Slope method is used to calculate each link’s traffic flow moving direction. Then extend flow direction of the link to a range by adding and subtracting an exogenous degree as the width of the range. Generally, most links go straight or turn gradually, an exogenous 20-degree angle is set during matching GPS points falling in a link. Fig 6 presents the instantaneous positions of taxis that were carrying passengers from 8:00am-9:00am on Jan. 12th, 2015 in the study area. Fig 6 distinguishes that most of the GPS data points focus on expressways and trunk roads, which shows the fact that traffic volume is usually much higher on these high grade level roads. In other words, it shows the potential of higher guidance utility on these roads. Besides, in reality, these may be bottlenecks where congestions are more likely to happen. Considering the limit of computational resources, only expressways and trunk roads are considered for VMS location allocation analysis, which results in 36 directional links presented in Fig 7. All these expressways and trunk roads in the study area are surrounded by auxiliary roads with exits and entrances. On average, entrances and exits appear every 1 kilometer, which allows drivers to easily divert from original route according to traffic information disseminated by VMS in road network.

**Fig 8:**
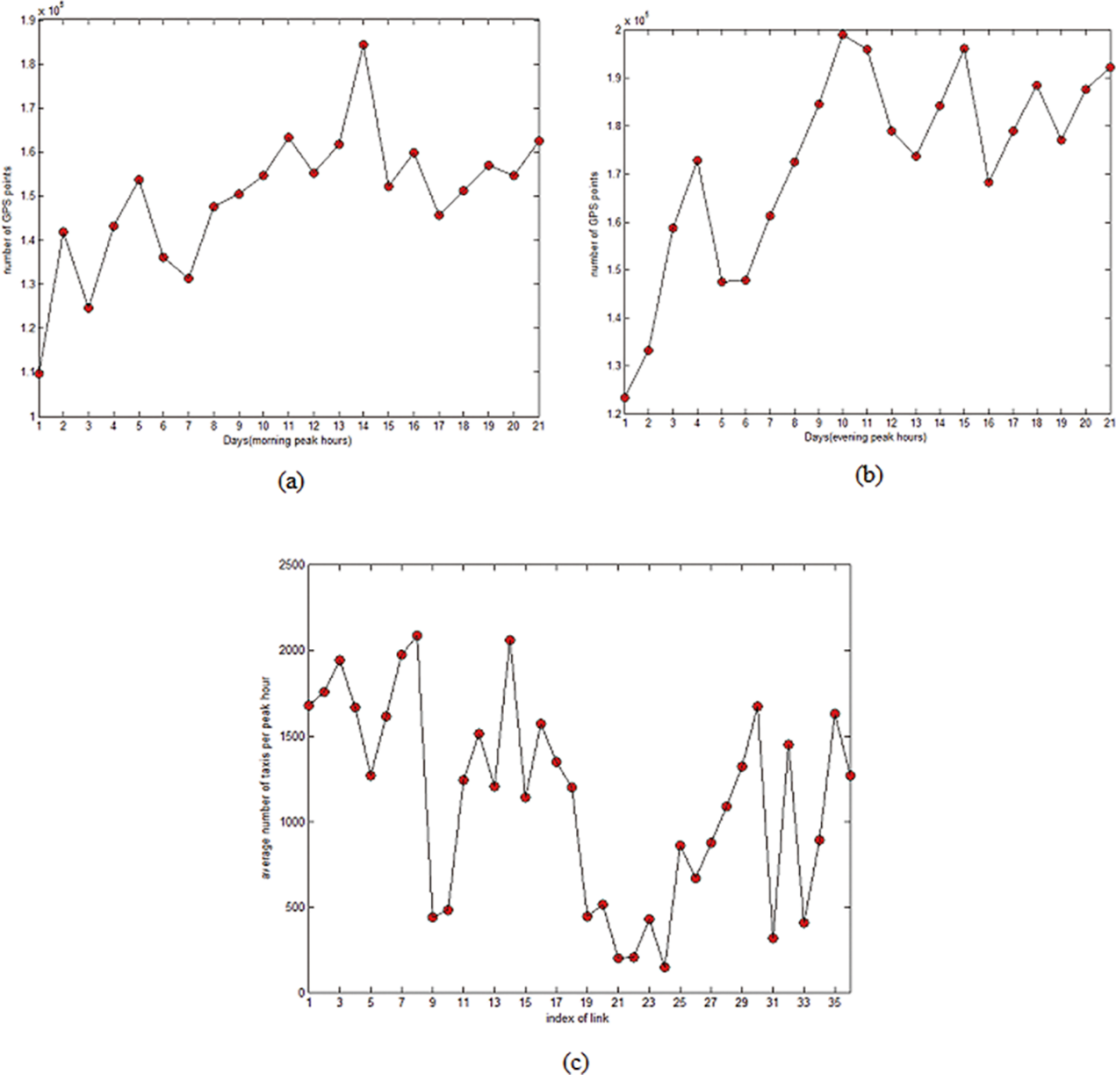
A glance of GPS records from taxis carrying passengers during peak hours in the study area: (a) morning peak hours; (b) evening peak hours; (c) average number of taxis with passengers per peak hour on each link.

In this study, the proposed VMS location allocation method is demonstrated by using taxis GPS data collected during peak hours on workdays in January 2015. As peak hours are more challenging to the urban road network, traffic information disseminated by VMS is more meaningful during peak hours. Only peak hours (7:00AM-9:00AM and 5:00PM-7:00PM) on workday are considered. Data of weekends and special holidays are excluded, which results in 42 peak periods in 21 workdays. Speed and traffic volume information can be obtained through statistics during matching GPS data with study area map. Fig 8(A) and Fig 8(B) present variation number of GPS records collected from taxis with passengers during morning and evening peak hours in the study area. On average, 149,571 and 172,520 GPS records are observed during morning peak and evening peak respectively. Fig 8(C) shows the average number of taxis with passengers per peak hour on each link in the road network. These amount of data are sufficient to support VMS location allocation analysis.

For distribution measurement of GPS probe vehicles in traffic flow, this is equivalent to throwing sands into a city randomly. The ordinary cars are black sand while the taxis with GPS devices are red sand. When this process ends, the distribution of black or red sand will be uniform. What’s more, the rationality of this uniform distribution assumption has been proved in the work of Andrew et al. 2014 [22]. As taxis proportion is uniformly distributed in the whole network [23–24], taxis number can be used to represent traffic flow to some extent.

### Quantification of guidance utility

In percentile speed calculation, *p* = 50 is adopted, which is the median speed. Speed threshold is used to determine congestion by checking if the speed is less than the threshold. Then traffic condition is transformed into binary variable representing congested or uncongested states in each time interval for each link. Time interval reflects time magnitude for data aggregation. Widening time interval may result in missing some useful information by artificially refer to and averagely aggregate more data in time. Thus, employing shorter time interval can make the percentile speed more close to traffic condition. However, if a very small time interval is used, the number of GPS points for each link at each time interval will be limited, which may not be enough to generate a reliable speed estimate to reflect the real traffic condition. In this study, the time interval is set as 5 minutes to aggregate instantaneous speed of GPS data points for each link. Then congestion is identified by critical speed threshold of 25 km/h. If time duration for congestion is short, this congestion is viewed as traffic transient status and not a real congestion state. Here, only congestion duration covers over two-times interval (10 minutes) is considered. The number of peak hours with congestion occurrence is counted for each link. The peak hour congestion probability for each link can be obtained through dividing the counters by the total number of the peak hours, referring to Eq (2) in subsection II.A. During all the peak hours, the flow volume and average congestion duration for each link are made statistics. Using Eq (3), the traffic information for each link can be calculated.

To analyze guidance utility and information coverage, the activation segment of VMS need to be determined. Chiu and Huynh [5] used 3.2 kilometers (2 miles) as VMS activation segment, which may differ in different countries and cities. Referring to reference [5] and advice of experienced experts, the VMS activation segment is set to be 4 kilometers. Based on traffic information attenuation Eq (4), through $\alpha^{l_{i}^{j}}\approx 0$ as $l_{i}^{j}=4$, the attenuation coefficient *α* is deducted to be 0.45. The distance from upstream link *i* to downstream link *j* is obtained by the shortest route distance algorithm. Through Eq (6), guidance utility for each link can be acquired. Data of index 1–10 links are listed in Table 1.

**Table 1:** Data of index 1–10 links in test area during peak hours.

link index	flow volume (vehicles per hour)	congestion probability	congestion duration (minutes)	length (km)	guidance utility
1	1,676	0.81	34	1.435	102,195
2	1,758	0.93	53	1.435	93,949
3	1,943	1.00	99	1.887	196,620
4	1,666	1.00	80	1.887	206,385
5	1,270	0.88	74	1.407	104,738
6	1,616	0.67	34	1.407	101,850
7	1,978	1.00	68	1.566	185,127
8	2,089	0.90	60	1.566	179,976
9	439	0.43	10	0.828	39,759
10	482	0.98	70	0.828	98,800

### Heuristic search algorithm to allocate location of VMS

According to the description of the developed heuristic search algorithm in subsection II.D, variables should be initialized first. As there is no VMS has been located in the road network, information redundancy *C*_*i*_ = 0 for ∀*i* ∈ **N**, *i* is index number of link, **N** is the set of all links in road network; *k* = 1 is traversal iteration number; the links set which have located VMS **A** = *ϕ*; the links set which have been searched is **S** = *ϕ*. Next, the guidance utility is classified into *a* classes through K-NN clustering algorithm. Here in order to demonstrate heuristic search algorithm, the value of *a* is set to be 4. One note should be pointed that different classification number *a* may lead to different location result, which will be discussed lately.

According to the algorithm developed in subsection II.D, the 4th link is firstly chosen as candidate link to locate a VMS. Then tabu search algorithm is conducted to look through if there is a better option in the neighborhood of link 4 to allocate the first VMS in the road network. According to the moving direction illustrated in Fig 3, the 29th link is the very upstream link of the 4th link. Through comparison, the 29th link is not in the same guidance utility class with the 4th link. So the 4th link is chosen as the first link to locate the first VMS. Add the 4th link to set **A** and **S**. Then the information coverage *C*_*i*_ for each link is updated based on Eq (7) in Step 3. The information coverage set **C** is classified into *b* classes through K-NN clustering algorithm. Here the value of *b* is set to be 4 and discussed lately with *a*. The information coverage class with minimum mean value is chosen as the candidate set. In the candidate set, the guidance utility is sorted in descending order. In this candidate set, the 3rd link has the largest guidance utility and is chosen as candidate link to locate VMS. Then the 3rd link’s neighborhood is searched by tabu search algorithm, and the very upstream links set {25, 10} are checked. The two upstream links are not in the same guidance utility class with the 3rd link. So the 3rd link is chosen as the final link to locate another VMS. Repeat location allocation process until the termination criterion in Section II.D Step 3 is met. As the heuristic search algorithm stops, a project schedule of the VMS installation process is **A** = {4,3,7,8,6,36,5,27} and the saturated number of VMS is 8 for the study area, which is shown in Table 2.

**Table 2:** The location allocation order of VMS as *a* = 4,*b* = 4.

link index	guidance utility	flow volume (vehicles per hour)	congestion probability	congestion duration (minutes)	length (km)	order
4	206,385	1,666	1.00	80	1.887	1
3	196,620	1,943	1.00	99	1.887	2
7	185,127	1,978	1.00	68	1.566	3
8	179,976	2,089	0.90	60	1.566	4
6	101,850	1,616	0.67	34	1407	5
36	131,613	1,269	0.95	65	1.686	6
5	104,738	1,270	0.88	74	1.407	7
27	51,458	877	0.50	37	1.451	8

For location allocation problem, there are many ways to quantify the effectiveness of the proposed method, such as a simulation with a before and after evaluation, profit and loss, drivers response, some quantitative indicators. Any measurement method can be chosen to evaluate the effectiveness of location allocation model. In this study, four indicators are selected to evaluate the effectiveness of the method: average guidance utility $\sum_{i}G_{i}b_{i}/\sum_{i}b_{i}$, links percentage $\sum_{j}u_{j}/N$ under benefit of VMS, and sum information redundancy $\sum_{i}(C_{i}-1)b_{i}$, saturated number of VMSs $\sum_{i}b_{i}$ in the road network.

**Fig 9:**
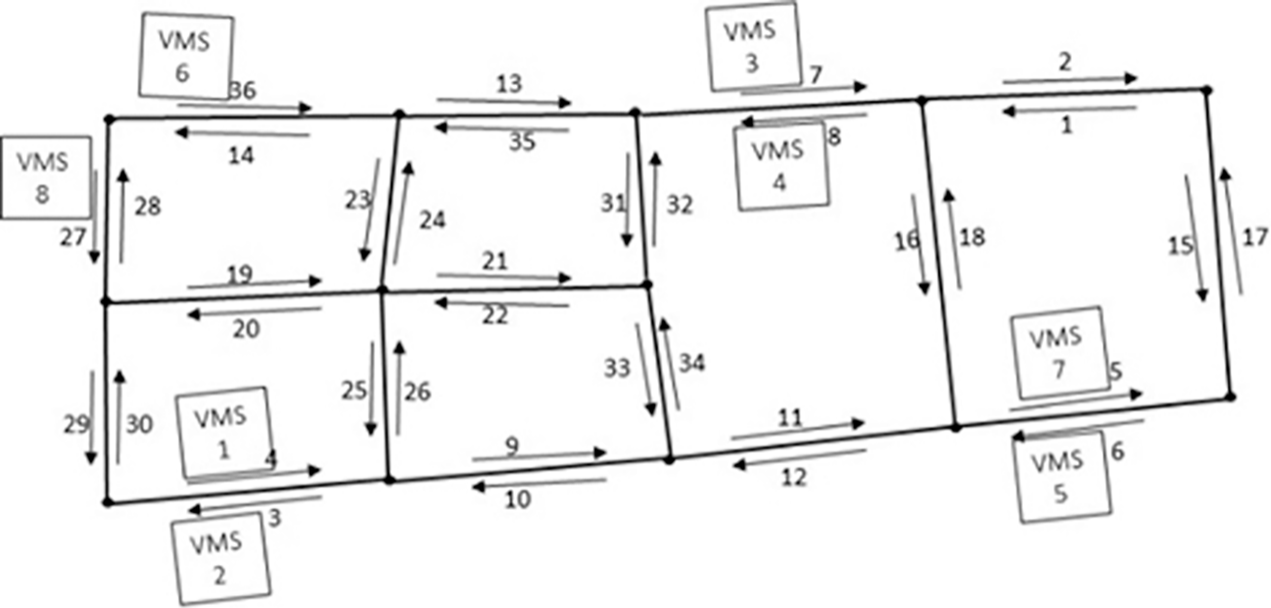
The project schedule of VMS installation process is {4,3,7,8,6,36,5,27}, and the saturated number of VMS is 8 in test area (number in square under VMS means installation precedence order).

In heuristic search algorithm, the classification number of guidance utility and coverage degree are important factors and should be set with an appropriate value. With a larger classification number, the guidance utility will be classified into more classes. For instance in Fig 3(A), if link 3 and link 4 are classified into different classes, link 4 will be chosen to install a VMS and link 3 can’t benefit from this VMS. Vice versa, if classification number is too small, the guidance utility will be classified into less classes. When classification number is small, link 3 and link 4 may be classified into the same class with link 2. Ideally, link 3 will be chosen as candidate link to locate VMS firstly. Because link 2 is in the same guidance utility class with link 3, according to the move direction described in Fig 3, link 2 will be chosen as the last link to install VMS. In fact, it is more meaningful to choose link 3 to install VMS. Table 4 shows the results of different classification number of guidance utility and information coverage. As the results of *a* = 4,*b* > 4 are same with *a* = 4,*b* = 4, and the results of *a* > 6,*b* = 4 are same with *a* = 6,*b* = 4, the remaining cases are presented in Table 3. It’s easy to find that condition *a* = 4,*b* = 4 and *a* = 6,*b* = 4 are much better than other kinds of classification on saturated number of VMSs, sum information redundancy and average guidance utility. For the percentage of links under benefit of VMS and sum information redundancy, condition *a* = 4,*b* = 4 is better than *a* = 6,*b* = 4; for sum information redundancy condition *a* = 4,*b* = 4 is better than *a* = 6,*b* = 4; for average guidance utility in the road network, there is a small but not obvious gap between the condition *a* = 6,*b* = 4 and *a* = 4,*b* = 4 as $\frac{144,721}{149,796}=0.966$. For the percentage of links under benefit of VMS, in condition *a* = 4,*b* = 4, only 1^st^ link is out of benefit of VMS; however, in condition *a* = 6,*b* = 4, there are 3 links {1,6,18} out of benefit of VMS. In the view of cost saving, it is acceptable for those links with low flow volume and congestion probability not in the benefit of upstream VMSs. As links set {6,18} have high flow volume, for percentage of links under benefit of VMS, condition *a* = 4,*b* = 4 is better than *a* = 6,*b* = 4. Based on above analysis, classification *a* = 4,*b* = 4 is better than *a* = 6,*b* = 4. From above analysis, it could be concluded that classification *a* = 4,*b* = 4 is best in a variety of classification schemes, and the corresponding VMSs installation precedence order and positions in road network are illustrated in Fig 9.

**Table 3:** Comparison of VMS location in different classification number of guidance utility *a* and information coverage *b*.

classification number	saturated number of VMSs	percentage of links under coverage	sum information redundancy	average guidance utility
a = 2,b = 4	14	100%	2.53	102,812
a = 3,b = 4	10	97.3%	0.91	123,158
a = 4,b = 4	8	97.3%	0.203	144,721
a = 5,b = 4	12	100%	1.399	117,192
a = 6,b = 4	8	91.7%	0.285	149,796
a = 4,b = 2	12	97.3%	1.608	113,475
a = 4,b = 3	9	97.3%	0.55	139,685

**Table 4:** Comparison of our algorithm with backtracking algorithm and field VMS.

algorithm	VMS location links set	average guidance utility	percentage of links in coverage	sum information redundancy
our algorithm	{4,3,7,8,6,36,5,27}	144,721	97.3%	0.203
backtracking algorithm	{8,14,2,1,35,6,16,18}	97,089	77.8%	2.05
Beijing Urban	{1,3,4,5,7,10,14,18,23,26,30,32,36}	103,070	94.4%	3.48

**Fig 10:**
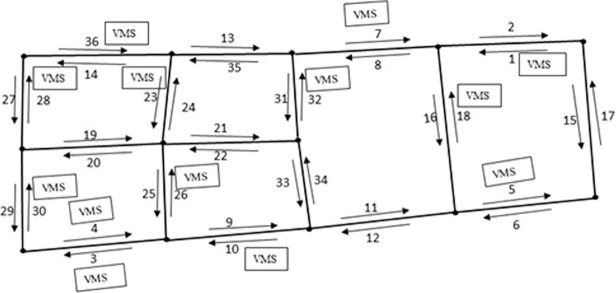
The filed VMS installation in test area.

The comparison would like to be conducted between this work and other ways to allocate location of VMS. As Variable Message Sign (VMS) has been applied in Beijing to release traffic information, a field study is conducted on the location and direction of the VMS in the test area, which is shown in Fig 10. As pointed out in section introduction, data limitation is one of the obstacles to the study of VMS location problem. Given the difficulty in obtaining other sources data that previously published methods need, other methods to locate VMS are not easy to be implemented. Backtracking algorithm is used in previous literatures [25] to optimally solve location allocation problem, backtracking algorithm to locate VMS can be given a try by scanning road grade, traffic flow, information intensity and attenuation. As backtracking algorithm can also show installation order of VMS, VMS location number is set to 8 for backtracking algorithm, which is the saturated number in our result. Comparison of our work with field located VMSs and backtracking algorithm is conducted and the comparison result is shown in Table 4. It shows that our method has more guidance utility, higher links coverage percentage, less information redundancy than field located VMS and backtracking algorithm. As our saturated VMSs number 8 is less than field VMSs 13, our method can save implementation cost compared with field installed VMS.

As Fig 10 illustrated, there are 13 VMSs installed in different road segments of the area, which are {1,3,4,5,7,10,14,18,23,26,30,32,36} denoted as *S*_*r*_. For our method, the project schedule of VMS installation is {4,3,7,8,6,36,5,27} and denoted as *S*_*p*_. After comparison, there are five VMSs {4,3,7,36,5} are consistent in both our method and the field VMSs. For the other eight field VMS locations {1,10,14,18,23,26,30,32}, they are either in the neighboring downstream link or upstream link of *S*_*p*_. For example, link 1 in *S*_*r*_ is not in *S*_*p*_, but it is in the upstream of link 8, which is included in *S*_*p*_. Compare to *S*_*r*_,*S*_*p*_ can provide higher average guidance utility by covering nearly same percentage of links with much less VMS devices and information redundancy, which is shown in Table 4.

During the calculation process, except the developed multi-objective optimization model and low-cost GPS probe data, our method don’t rely on the assistance of any other additional tools or subjective judgment from practitioners. For the test area, in our work, the project schedule of the VMS installation process is {4,3,7,8,6,36,5,27} and the saturated number of VMS is 8 for the study area, which is shown in Fig 9 and Table 2. It can be seen that our method gives not only saturated number of VMSs, but also the installation precedence order in a road network, which is one advantage of our method. As the saturated number of VMS is 8 in the test area, which means redundancy occurs if any the 9th VMS is added to this test area. The output of saturated number of VMSs can effectively avoid wasting money to install two close VMSs to provide information with redundancy, which also saves implementation cost. During the implementation process of the algorithm, the more profitable location of VMS will be selected in priority. If financial support is not sufficient to cover all the VMSs, VMSs installation can be divided into different stages referring to the installation precedence order shown in the last column in Table 2. The case study in Beijing urban road network using taxis as probe vehicles shows the effectiveness of this method.

## Conclusion

This paper proposes a multi-objective optimization model to allocate the locations of VMS by maximizing average guidance utility of VMSs and number of benefited links while minimizing information redundancy. Each objective has solid physical meaning: maximizing guidance utility is to ensure each VMS can benefit most drivers in the road network, which is derived from the passively collected GPS probe data and physical topology of the road network. Minimizing information redundancy is physically represented as lowering mutual impairing between any two VMSs and save implementation cost without artificially set budgetary constraints. Maximizing number of benefited links of VMS in the road network is to disseminate as many as possible links’ information to upstream drivers through VMS.

As discussed earlier, data limitation is one of the obstacles to study the VMS location problem. Different from previous methods depending on subjective judgment, simulation or other data sources, this work fully utilizes passively collected GPS data to allocate the locations of VMS, without the assistance of any other additional tools or using subjective judgment from practitioners. The proposed multi-objective optimization model can be applied to both urban roads and highway networks with enough GPS data covering the network.

The developed heuristic search algorithm can calculate the saturated number of VMS for a road network and optimize the project schedule of VMS installation process based on the proposed objectives. If there is no enough money to implement all the VMSs at the same time, VMSs can be installed in different stages.

The methodology presented in this paper relies on multi-objective optimization model and accurate real-world data to allocate the locations of VMS facilities. However, the interaction between guidance information and traffic flow dynamics is not taken into consideration. Future research should consider mutual feedback mechanism between actual road conditions and VMS guidance information.
